# Multi-Scale Capsule Attention Network and Joint Distributed Optimal Transport for Bearing Fault Diagnosis under Different Working Loads

**DOI:** 10.3390/s21196696

**Published:** 2021-10-08

**Authors:** Zihao Sun, Xianfeng Yuan, Xu Fu, Fengyu Zhou, Chengjin Zhang

**Affiliations:** 1 School of Mechanical Electrical and Information Engineering, Shandong University, Weihai 264209, China; sunzihao@mail.sdu.edu.cn (Z.S.); fuxu@mail.sdu.edu.cn (X.F.); cjzhang@sdu.edu.cn (C.Z.); 2 School of Control Science and Engineering, Shandong University, Jinan 250100, China; zhoufengyu@sdu.edu.cn

**Keywords:** intelligent fault diagnosis, domain adaptation, multi-scale capsule attention network, joint distribution optimal transport, different working loads

## Abstract

In recent years, intelligent fault diagnosis methods based on deep learning have developed rapidly. However, most of the existing work performs well under the assumption that training and testing samples are collected from the same distribution, and the performance drops sharply when the data distribution changes. For rolling bearings, the data distribution will change when the load and speed change. In this article, to improve fault diagnosis accuracy and anti-noise ability under different working loads, a transfer learning method based on multi-scale capsule attention network and joint distributed optimal transport (MSCAN-JDOT) is proposed for bearing fault diagnosis under different loads. Because multi-scale capsule attention networks can improve feature expression ability and anti-noise performance, the fault data can be better expressed. Using the domain adaptation ability of joint distribution optimal transport, the feature distribution of fault data under different loads is aligned, and domain-invariant features are learned. Through experiments that investigate bearings fault diagnosis under different loads, the effectiveness of MSCAN-JDOT is verified; the fault diagnosis accuracy is higher than that of other methods. In addition, fault diagnosis experiment is carried out in different noise environments to demonstrate MSCAN-JDOT, which achieves a better anti-noise ability than other transfer learning methods.

## 1. Introduction

Industrial mechanical systems are developing in the direction of complexity, precision and integration, and the tightness of mechanical equipment is increasing [1]. Therefore, operation state monitoring for mechanical equipment is becoming increasingly important. A bearing is the core component of rotating mechanical equipment, and its ability to operate is very important. Once the bearing fails, it will not only affect the normal operation of mechanical equipment but also cause serious or irreparable accidents and threaten the safety of personnel and property. As a popular fault diagnosis method, data-driven intelligent fault diagnosis has attracted a large number of researchers’ attention in recent years [2]. Using a large volume of fault samples, data-driven fault diagnosis methods can learn the knowledge implicit in the data, and they are particularly effective for the complicated systems in which it is otherwise difficult to obtain accurate mathematical system models [3]. With the rapid development of intelligent mechanical systems, data can be collected at a high speed, which brings both industry and academia new opportunities and challenges [4]. Hence, it is meaningful to find an intelligent bearing fault diagnosis method with better performance.

Traditional machine learning fault diagnosis methods, for instance, decision tree, random forest [5] and support vector machines (SVMs) [6], require a complex manual feature extraction and selection process, which has a significant effect on the diagnosis accuracy. In recent years, the application of deep neural networks [7] in the wide field of fault diagnosis has gradually increased. Through an end-to-end method, the deep network-based method can avoid the manual feature extraction and selection process, which is time-consuming and overly dependent on experience. As a typical deep learning algorithm, convolutional neural networks (CNNs) are widely adopted in the broad area of fault diagnosis. For example, in [8], Wen et al. proposed a novel CNN algorithm based on the well-known Lenet-5 model for bearing fault diagnosis. In this CNN algorithm, one-dimensional raw vibration signals are skillfully processed and converted into two-dimensional grayscale images by signal superposition, and then the obtained grayscale images are fed into a CNN, which is used for fault diagnosis. Han et al. [9] presented a hybrid fault diagnosis framework, which mainly contains two parts: one part is a spatial-temporal pattern network, which is focused on the task of spatial–temporal feature learning, and the other part is a CNN, which is devoted to conditional classification. Zhang et al. [10] presented a well-designed CNN model, in which the first layer has wide convolution kernels, and the one-dimensional raw vibration signals are fed into the proposed CNN model. Experimental results indicated that the well-designed CNN model have good anti-noise performance in fault diagnosis. In addition, many CNN-based fault diagnosis methods using two-dimensional time-frequency image representations have also been exploited, such as [11]. To fully exploit the advantages of the well-trained CNN model in feature learning, the core idea of these methods is to convert one-dimensional time domain original training samples into time-frequency images for training network. 

Compared with the fault diagnosis approaches based on classical shallow learning models, deep network-based methods show superior performance. Assuming that the data acquisition processes of training and testing sets are conducted under the same working condition, i.e., the data distribution of training and testing set is consistent, the majority of deep network-based fault diagnosis methods are effective. However, this strict assumption is nearly impossible in practical applications. For rolling bearings, the data distribution will change when the working load or rotating speed changes. To address this issue, transfer learning is an attractive alternative, which bring us a new perspective. In [12] and [13], Yan et al. proposed new fault diagnosis methods based on transfer learning, which promotes the research and application of transfer learning in the area of fault diagnosis significantly. In addition, extensive fault diagnosis experiments were conducted and experimental results indicated that the presented methods achieved impressive and promising performance.

As a representative and widely adopted transductive transfer learning method, domain adaption (DA) technique could align features distribution between the target domain and source domain during the training procedure while maintaining a good classification result. For example, using maximum mean discrepancy (MMD) and multi-kernel model, An et al. [14] presented a fault diagnosis framework, which achieved a high diagnosis accuracy. In [15], a new partial adversarial DA fault diagnosis approach was presented based on stacked auto-encoder. Using MMD and domain adversarial training (DAT), Li et al. [16] presented a novel diagnosis scheme, which achieved enhanced feature representation ability, and the ensemble learning was adopted to obtain the final diagnosis result. Wen et al. [17] proposed a new deep transfer model-based diagnosis approach, in which, the feature extraction task is fulfilled by a sparse auto-encoder network, and the inconsistency between the distributions of testing and training set is minimized by the MMD, thereby the domain adaptation process is accomplished. Li et al. [18] presented an end-to-end scheme that combines bidirectional signals and capsule networks to input horizontal and vertical vibration signals into the neural network. Using the proposed scheme, domain-invariant features can be learned from training samples collected under variable working conditions. To minimum the distribution differences across domains, Chen et al. [19] presented a well-designed transfer network-based multi-domain diagnosis scheme, which integrates a task-specific encoder network and DAT. In [20], based on the multi-scale multi-domain feature, an improved diagnosis scheme was designed, which is effective in dealing with the fault diagnosis problems under polytrophic working conditions. In order to extract domain-invariant features from the raw signals, Wang et al. [21] presented a deep adversarial domain adaptation network (DADAN), which uses DAT and the Wasserstein distance. By embedding the useful discriminative knowledge in the label predictions into the domain classifier, Yu et al. [22] proposed a powerful conditional DADAN, which can align the features distribution between the target and source domains better. In addition, a new loss function is introduced to better extract invariant and discriminative features. Li et al. [23] proposed a novel multi-layer domain adversarial graph convolutional network (DAGCN), which uses graph convolution to extract features, and uses domain adversarial training and maximum mean discrepancy to minimize distribution differences of target and source domain features. Huang et al. [24] presented a new promising deep adversarial capsule network (DACN), which can not only separate the composite fault into several single faults intelligently, but also generalize faults under certain working conditions into faults under other new working conditions. Using optimal transport (OT), Liu et al. [25] presented a novel diagnosis approach based on deep DA model. First, an improved auto-encoder network was used to learn class discrimination features. Second, domain-invariant features are extracted by minimizing OT cost function between target and source domains. Finally, the offline trained classifier is tested with the target domain samples. The results indicated that these methods achieve better fault diagnosis accuracy and domain adaptability. Compared with previous deep learning methods without domain adaptation, the methods with domain adaptation can maintain a good fault diagnosis effect when the working conditions change. Thus, domain adaptation is an effective method for fault diagnosis under different working conditions. However, the above methods usually consider fault diagnosis in the case where only small working condition changes occur without considering the influence of noise on fault diagnosis.

In this article, to further facilitate the accuracy of bearing fault diagnosis under different loads as well as the anti-noise ability of the fault diagnosis model, a transfer learning-based method using multi-scale capsule attention network and joint distribution optimal transport (MSCAN-JDOT) is proposed. The main contributions are summarized as follows:

- A new transfer learning-based fault diagnosis approach called MSCAN-JDOT is proposed which accepts raw vibration signal as input and can effectively perform end-to-end fault diagnosis without the time-consuming and experience-dependent manual feature extraction.
- The proposed MSCAN-JDOT adopts multi-scale capsule attention networks as feature extraction networks, which can better extract fault features, and uses joint distribution optimal transport for domain adaptation, which can effectively align the fault features under different loads.
- MSCAN-JDOT achieves high accuracy and strong anti-noise performance for bearing fault diagnosis under different working loads.

The rest of this article is organized as follows. Section 2 briefly introduces the theory of capsule networks and optimal transport. Section 3 describes the proposed MSCAN-JDOT in detail. Section 4 evaluates the performance of MSCAN-JDOT on the rolling bearing dataset. Section 5 concludes this article.

## 2. Capsule Network and Optimal Transport

### 2.1. Capsule Network

**Figure 1:**
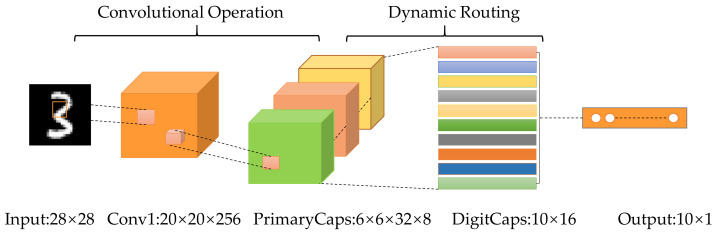
Architecture of a capsule network with three layers.

In the traditional CNN [26], features are transferred to the next layer through a pooling operation. The regional maximum or regional average is selected through max pooling or mean pooling. However, the spatial information will be lost inevitably in the process of the pooling operation. To overcome this problem, Sabour et al. [27] presented the capsule network. Capsule networks use capsules instead of neurons in the traditional neural network to extract invariant features more effectively. The capsule is a vector consisted of a certain number of neurons, and each neuron indicates a certain attribute of a particular instance, such as angle, color, and other properties [27]. After that, several improved capsule networks have been proposed. For example, Hinton et al. [28] proposed a matrix capsule network with EM routing algorithm. Ribeiro et al. [29] proposed a capsule routing algorithm based on Variational Bayes, which improved the routing mechanism of capsule network. Similar to other neural networks, the capsule network is also composed of multi-layer networks, primarily including the convolutional layer, primary capsule layer and digit capsule layer. Taking MNIST handwritten digit classification as an example, a simple three-layer capsule network is illustrated in Figure 1. The first convolution layer extracts the input image into a feature map, which serves as the input to the primary capsule layer. Second, the primary capsule layer extracts the low-level features and divides them into capsules with dimensions of 8. Then, the digit capsule layer obtains the output capsules from the primary capsule layer through dynamic routing and converts them into capsules with dimensions of 16. Finally, the capsules are classified using other layers and classifiers.

In capsule network, the length of the output capsule vector represents the probability of the existence for the instance category. Therefore, when the output category is consistent with the label, the output capsule has a long instantiation vector. Each digital capsule has a separate margin loss:

(1) $$L_{c}=T_{c}\max{\left(0,m^{+}-\left\|v_{c}\right\|\right)}^{2}+\lambda \left(1-T_{c}\right)\max{\left(0,\left\|v_{c}\right\|-m^{-}\right)}^{2}$$

where c is the classification category, $T_{c}$ is the indicator function of the classification, $T_{c}=1$ when category c exists, $T_{c}=0$ when category c does not exist. λ is the trade-off coefficient, $m^{+}$ is the upper boundary of classification probability and $m^{-}$ is the lower boundary of classification probability. In addition, $\left\|v_{c}\right\|$ is the $L_{2}$ distance of the vector $v_{c}$. Since each category of capsule has a separate margin loss $L_{c}$, the total margin loss is the sum of $L_{c}$ for all categories.

### 2.2. Optimal Transport

Optimal transport [30] (OT) is a method that can be used to compare probability distributions in a geometrically reasonable way. OT studies the empirical distribution and makes use of the geometric structure of the data embedding space. According to Equation (2), OT searches for a probabilistic coupling $\gamma \in \Pi \left(\mu_{1},\mu_{2}\right)$ between two distributions $\mu_{1}$ and $\mu_{2}$, which finds a minimal transport cost:

(2) $$OT_{c}\left(\mu_{1},\mu_{2}\right)=\underset{\gamma \in \Pi \left(\mu_{1},\mu_{2}\right)}{\inf}\int_{R^{2}}c\left(x_{i},x_{j}\right)d\gamma \left(x_{i},x_{j}\right)$$

In the discrete case, this becomes:

(3) $$OT_{c}\left(\mu_{1},\mu_{2}\right)=\min_{\gamma \in \Pi \left(\mu_{1},\mu_{2}\right)}<\gamma ,C{>}_{F}$$

where $x_{i},x_{j}$ belong to $\mu_{1},\mu_{2}$, respectively, the cost function $c\left(x_{i},x_{j}\right)$ measures the difference between $x_{i}$ and $x_{j}$, C is the cost matrix composed of $c\left(x_{i},x_{j}\right)$, and $\Pi \left(\mu_{1},\mu_{2}\right)$ describes the joint probability distribution of $\mu_{1},\mu_{2}$.

Optimal transport has been used as a common method that situates the source distribution and target distribution closer to each other by finding a transmission probability coupling matrix γ between two different distributions to minimize the cost matrix C. Moreover, experiments demonstrate that better constraint of the structure of γ using entropy or regularization terms contributes to better empirical results [31].

## 3. Proposed Method

**Figure 2:**
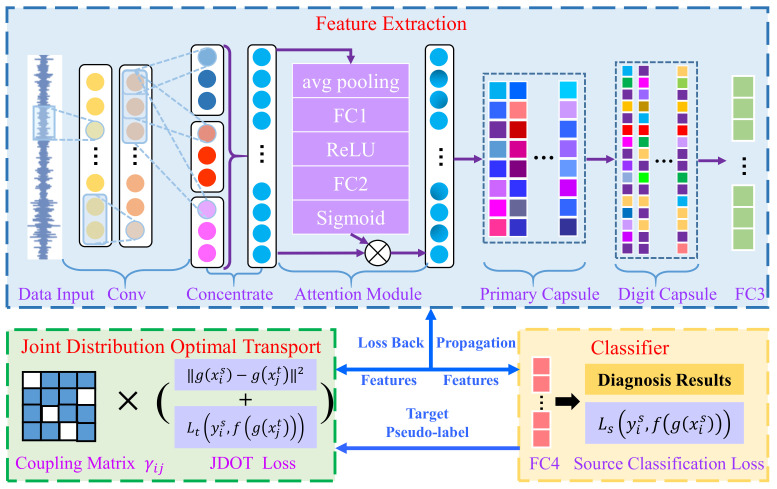
Architecture of the MSCAN-JDOT.

In this article, to improve fault diagnosis accuracy under different loads as well as improve the anti-noise performance of the model, an intelligent fault diagnosis approach based on multi-scale capsule attention network and joint distribution optimal transport is proposed. Figure 2 shows the architecture of MSCAN-JDOT, which primarily contains four components: data input, feature extraction, classifier and domain adaptation. First, in the data input component, the model accepts a one-dimensional original sample as data input without any manual feature extraction. The labeled samples under one load are used as the source domain and the unlabeled samples under other loads are used as the target domain. The second component, feature extraction, includes a multi-scale convolution layer, an attention module, a primary capsule layer, a digit capsule layer and a fully connected layer. Then, in the classifier component, the fully connected layer transforms the feature dimensions and the fault diagnosis results are obtained using softmax. Finally, in the domain adaptive component, the adaptability of the model under different loads is implemented using joint distribution optimal transport.

### 3.1. Feature Extraction Details

In feature extraction, a multi-scale capsule attention network is proposed. First, three one-dimensional convolution layers are applied to directly learn the feature representation from one-dimensional raw signals. The first convolution layer uses wide convolution kernels. Wide convolution, on the one hand, can expand the receptive field of convolution operation and accelerate the speed of model training; on the other hand, wide convolution can enhance the anti-noise ability of the model. The second convolution layer uses small size kernels to enhance the local feature extraction ability. The convolution process of the first two layers is described as follows:

(4) $$y_{k}=\sigma (w_{k}⊗x+b_{k})$$

where $y_{k}$ is the output of the *k*th layer, $w_{k}$ and $b_{k}$ represent the weights and bias of the convolutional process, x represents the input of the convolution layer, ⊗ indicates the convolutional calculation, and σ is the ReLU activation function. To better extract the domain-invariant features of fault data, multi-scale convolutional layer, which can be described as Equation (5), is added in the third layer of the model:

(5) $$y_{ms}=concentrate(y_{31},y_{32},y_{33})$$

where $y_{31},y_{32},y_{33}$ are convolution outputs with convolution kernels of 3, 8 and 16, respectively, $y_{ms}$ is the output of multi-scale convolution layer, and concentrate(⋅) indicates splicing by channel.

Second, a channel attention module, which can focus on more meaningful input channels, is added after the multi-scale convolution layer. To calculate channel attention effectively, average pooling is adopted to compress the spatial dimension of input features. Then, the full connection layer and sigmoid is used to calculate the attention weight on the channel. Finally, the attention weight is multiplied by the corresponding channel to obtain the input features with attention. The process can be described as follows:

(6) $$y_{a}=x_{a}\cdot Sigmoid(W_{a_{1}}\cdot (\sigma (W_{a_{2}}\cdot pool(x_{a})+b_{a_{2}}))+b_{a_{1}})$$

where $x_{a}$ is the input of the attention module, $y_{a}$ is the output of the attention module, $W_{*}$ and $b_{*}$ are the weight and deviation of the full connection layer.

Third, a primary capsule layer and a digit capsule layer are added after the attention module, because the capsule network can extract various attributes of samples and better express the data features. The primary capsule layer can be described as follows:

(7) $$y_{conv}=\sigma \left(W_{conv}⊗x_{caps}+b_{conv}\right)$$

(8) $$y_{pcaps}=\sigma \left(W_{pcaps}⊗y_{conv}+b_{pcaps}\right)$$

where $x_{caps},y_{conv}$ are the corresponding input of the primary capsule layer, $y_{pcaps}$ is the output of the primary capsule layer, $W_{*}$ and $b_{*}$ are the weights and bias in the corresponding layer.

Aiming to build the relationships between two capsule layers, dynamic routing algorithm, which is displayed in Algorithm 1, is introduced between the primary and digit capsule layers. In Algorithm 1, $b_{ij}$ is the bias coefficient from capsule i in *l*th layer to capsule j in the next layer, and it is initialized to zero before algorithm iteration. $u_{j\left|i\right.}$ is the intermediate prediction vector between the ith capsule and the jth capsule, and it is equal to the multiplication of $u_{i}$ and the weight coefficient matrix $W_{ij}$. $c_{ij}$ is the weight coefficient of the intermediate prediction vector $u_{j\left|i\right.}$. $b_{i}$ and $c_{i}$ is the set of $b_{ij}$ and $c_{ij}$. The squash function is similar to the activation function in the convolutional neural network that carries out nonlinear transformation on the input vector and compacts the input vector to [0, 1]:

(9) $$v_{j}=\frac{{\left\|s_{j}\right\|}^{2}}{1+{\left\|s_{j}\right\|}^{2}}\frac{s_{j}}{\left\|s_{j}\right\|}$$

where $s_{j}$ is the input to the squash function and $v_{j}$ is the output of the squash function.
**Algorithm 1** Dynamic routing algorithmProcedure routing $\left(u_{j\left|i\right.},r,l\right)$ for all capsule i in layer l and capsule j in layer l+1: $b_{ij}\leftarrow 0$. for r iterations do  for all capsule i in layer l: $c_{i}\leftarrow softmax(b_{i})$  for all capsule j in layer l+1: $s_{j}\leftarrow \sum_{i}c_{ij}u_{j\left|i\right.}$  for all capsule j in layer l+1: $v_{j}\leftarrow squash(s_{j})$  for all capsule i in layer l and capsule j in layer l+1: $b_{ij}\leftarrow b_{ij}+u_{j\left|i\right.}\cdot v_{j}$ return $v_{j}$

The dynamic routing adopts an iteration number of 3, which was shown to be effective in [27]. In the first iteration, because $b_{ij}$ is set to 0, all intermediate prediction vectors $u_{j\left|i\right.}$ share the same weight coefficient $c_{ij}$. As the iteration proceeds, the intermediate prediction vector $u_{j\left|i\right.}$, which is more similar to the high-level capsule $v_{j}$, has a larger weight coefficient $c_{ij}$. This coefficient ultimately ensures that the features of the low-level capsule are more likely to be transferred to a similar, high-level capsule. After dynamic routing, the output $y_{dcaps}^{j}$ of digit capsule layer can be described as follows:

(10) $$y_{dcaps}^{j}=v_{j}=\frac{{\left\|s_{j}\right\|}^{2}}{1+{\left\|s_{j}\right\|}^{2}}\frac{s_{j}}{\left\|s_{j}\right\|}$$

Finally, the result of feature extraction is obtained through a full connection layer. The model parameters of MSCAN-JDOT are shown in Table 1.

### 3.2. JDOT Domain Adaptation

Courty et al. [31] presented the well-known joint distributed optimal transport (JDOT) method to avoid two-step adaptation (i.e., first performing domain feature adaptation and then learning the classifier from the adaptive features) by diametrically learning the classifier embedded in the cost function c. The goal of JDOT is to align the joint distribution of data features and labels, rather than just aligning the feature distribution. In the domain adaptive task, it is assumed that $\left(x_{i}^{s},y_{i}^{s}\right)$ and $\left(x_{j}^{t},y_{j}^{t}\right)$ are samples from the source and target domain, respectively. The JDOT cost function consists of the following two parts:

(11) $$d\left(x_{i}^{s},y_{i}^{s};x_{j}^{t},y_{j}^{t}\right)=\alpha c\left(x_{i}^{s},x_{j}^{t}\right)+\lambda_{t}L\left(y_{i}^{s},y_{j}^{t}\right)$$

where $c\left(x_{i}^{s},x_{j}^{t}\right)$ is the cost function that aligns the feature distribution, $L\left(y_{i}^{s},y_{j}^{t}\right)$ is the cost function that aligns the label distribution, and *α* and $\lambda_{t}$ are two coefficients that weight the cost of the two parts. Usually, the target domain label $y_{j}^{t}$ is unknown and the pseudo-label $f\left(g\left(x_{j}^{t}\right)\right)$ is generated using the classifier f and feature extractor g. Therefore, the JDOT objective function becomes:

(12) $$\underset{f,\gamma \in \Pi \left(\mu_{1},\mu_{2}\right)}{\inf}\int_{R^{2}}\alpha c\left(x_{i}^{s},x_{j}^{t}\right)+\lambda_{t}L\left(y_{i}^{s},y_{j}^{t}\right)d\gamma \left(x_{1},x_{2}\right)$$

In the discrete case, the objective function becomes:

(13) $$\min_{f,\gamma \in \Pi \left(\mu_{s},\mu_{t}\right)}<\gamma ,D_{f}{>}_{F}$$

where $D_{f}$ is the set of $d\left(x_{i}^{s},y_{i}^{s};x_{j}^{t},y_{j}^{t}\right)$.

The proposed MSCAN-JDOT method aligns the features distribution of source domain and target domain using joint distribution optimal transport; additionally, the label distribution is considered while the feature distribution is aligned. The objective function of MSCAN-JDOT can be described as:

(14) $$\min_{\gamma ,f,g}\frac{1}{n^{s}}\sum_{i}L_{s}\left(y_{i}^{s},f\left(g\left(x_{i}^{s}\right)\right)\right)+\sum_{i,j}\gamma_{ij}\left(\alpha {\left\|g\left(x_{i}^{s}\right)-g\left(x_{j}^{t}\right)\right\|}^{2}+\lambda_{t}L_{t}\left(y_{i}^{s},f\left(g\left(x_{j}^{t}\right)\right)\right)\right)$$

where $L_{s}\left(y_{i}^{s},f\left(g\left(x_{i}^{s}\right)\right)\right)$ is the source classification loss, ${\left\|g\left(x_{i}^{s}\right)-g\left(x_{j}^{t}\right)\right\|}^{2}$ and $L_{t}\left(y_{i}^{s},f\left(g\left(x_{j}^{t}\right)\right)\right)$ is feature alignment loss and label alignment loss between the source domain and target domain, γ is the coupling matrix, f is the classifier, g is the feature extractor, and α, $\lambda_{t}$ are two coefficients weighting the loss of the two parts. The parameters α and $\lambda_{t}$ are set as α=0.001 and $\lambda_{t}=0.0001$ according to [32].

### 3.3. General Procedure of the Proposed Method

**Figure 3:**
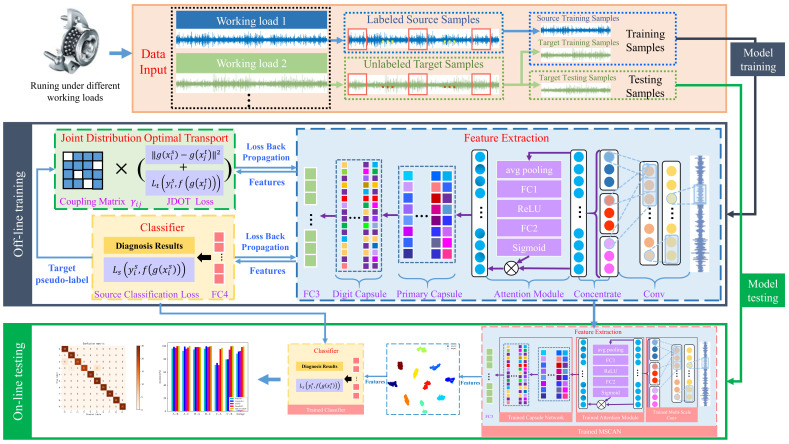
Flowchart of the MSCAN-JDOT.

The flowchart is shown in Figure 3 and the general procedures of the proposed MSCAN-JDOT are summarized as follows:

1. Data Input: In this step, the raw data sampled under different working loads are split into target domain and source domain. The training sets contains the labeled source domain samples and the unlabeled target domain samples, while the testing sets only contains the unlabeled target samples.
2. Training Stage: In this step, the training samples are input to the feature extraction network, and then the domain adaptation aligns the features of the source domain and target domain. Through the source prediction labels and target pseudo-labels generated by the classifier, the whole loss function of MSCAN-JDOT can be calculated by Equation (13). Finally, the model parameters can be updated with backward propagation.
3. Testing Stage: In this step, testing samples are used to validate the performance of the MSCAN-JDOT, which is well trained after sufficient epochs. In this stage, the network only carries out forward propagation without backward propagation. The model is evaluated by label prediction results and features alignment effect.

## 4. Experimental Analysis

In the actual operation of rolling bearings, the load and bearing speed will inevitably change. This section evaluates the proposed MSCAN-JDOT model under different loads. Since noise is inevitable in actual working environment and vibration signals are easily disturbed by noise, the anti-noise performance of the MSCAN-JDOT model is also evaluated. 

### 4.1. Dataset Introduction and Dataset Split

#### 4.1.1. Dataset Introduction

**Figure 4:**
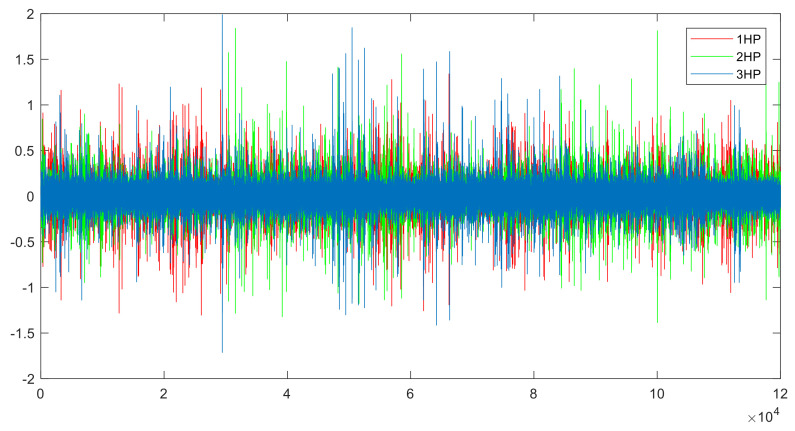
Drive-end data of 0.014_Ball under three different loads.

This article uses the Case Western Reserve University (CWRU) dataset [33] as the experimental data. An accelerometer is used for data collection. The sampling frequency is 12 kHz, and the sampling time is 10 s. Each dataset has 120,000 sampling points. This experiment uses three different load datasets, as shown in Table 2. A, B, and C represent 1, 2, and 3 loads, respectively, and the speed gradually decreases as the load increases. For each load, there are three different fault diameters and three different fault locations, for a total of nine different fault types. Each fault dataset contains three sets of data from different sampling locations, including driver-end data, fan-end data, and basic data. Table 3 shows the label assignment of the nine fault data and normal data. Figure 4 shows the drive-end data of 0.014_Ball under three different loads, and the data distribution changes significantly under different loads.

#### 4.1.2. Dataset Split

**Figure 5:**
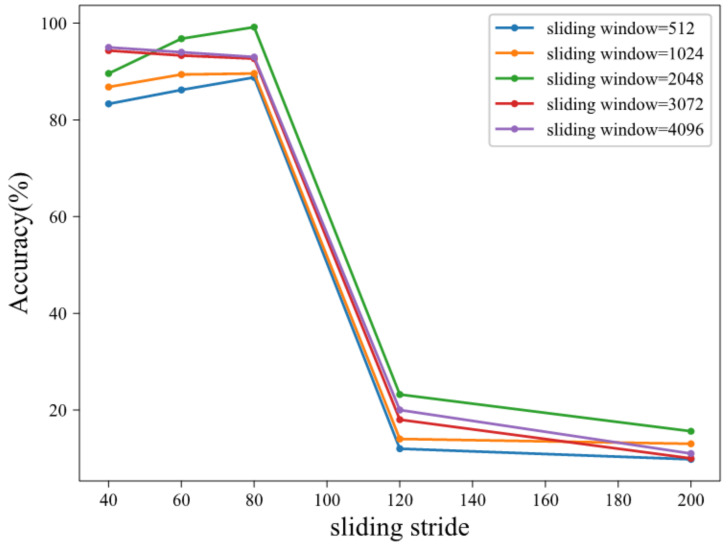
Sliding window length and stride parameter selection.

**Figure 6:**
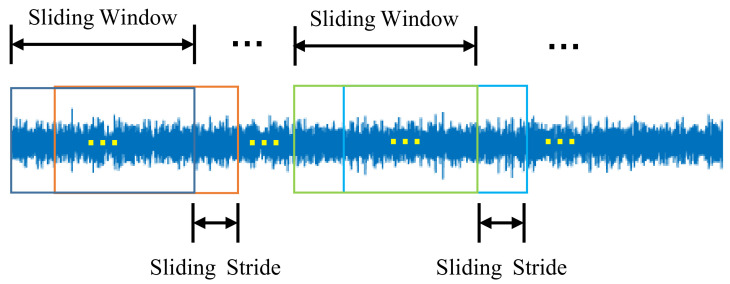
Data split.

Each original dataset contains 120,000 sampling points, which are separated into two groups with the same size. Because the number of samples in the training set is small, overlapping sampling is adopted for data splitting. The length and stride of sliding window affect the representation ability of fault attributes, so it is important to select the appropriate length and stride. As can be seen from Figure 5, the sliding stride has a great influence on the effectiveness of the method. With the increase in stride, the accuracy increases at first and then decreases. When the sliding stride is larger than 80, the performance of the method decreases sharply. Because the total number of training samples is small when the stride is too large, the effect of overlapping sampling is poor. The effect of sliding window length on the performance of this method is relatively small. When the length of sliding window is 2048, the overall performance is better. Therefore, the sliding window length is set to 2048 and the sliding stride is set to 80. Overlapping sampling is not adopted for the test set, and the length of each data sample is also 2048 sampling points. The data split is shown in Figure 6. Finally, 660 training samples and 25 test samples generated during each fault data collection step are selected. There are a total of 19,800 training samples and 750 test samples. In this article, each input data sample selects driver-end and fan-end data. Therefore, the input data dimension is (2048, 2). Before neural network training, input data need to be normalized:

(15) $$x^{*}=\frac{x-\mu }{\sigma }$$

where μ and σ is the sample mean and deviation, respectively.

### 4.2. Experimental Results and Performance Analysis

#### 4.2.1. Fault Diagnosis Experiments under Different Loads

In this part of the experiment, the source domain data with labels and the target domain data without labels are used as input, and the trained model is evaluated on the test set from the target domain without labels. According to the datasets and data split methods of three different loads, six experiments are used to test the MSCAN-JDOT model, including A-B, A-C, B-A, B-C, C-A, and C-B. For example, A-B represents that these experimental models are trained using dataset A with labels and dataset B without labels and tested with dataset B.

**Figure 7:**
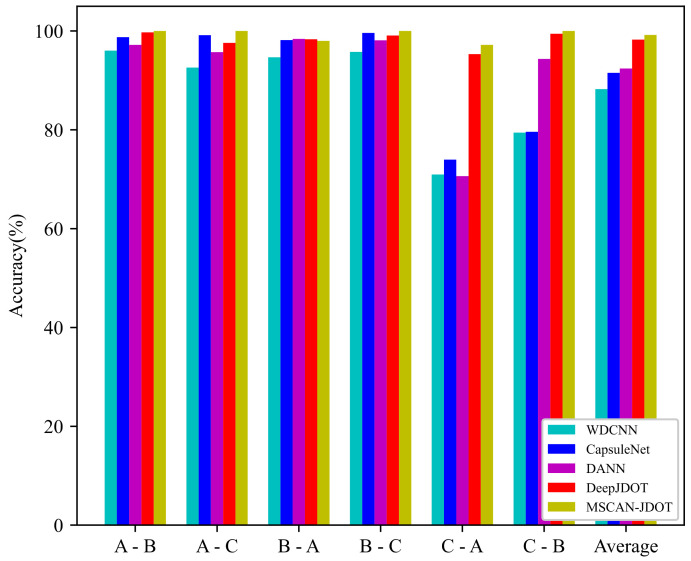
Visualization of transfer results on CWRU dataset.

To prove the effectiveness of the proposed MSCAN-JDOT, three widely applied algorithms are selected as competitors: the WDCNN [10], CapsuleNet, DANN [34], and DeepJDOT [32]. The WDCNN is the first layer wide convolutional kernel convolutional neural network. The CapsuleNet uses a one-dimensional capsule network for feature extraction, and the parameters are the same as those of capsule network in MSCAN-JDOT. The DANN is a domain adversarial neural network in which a 1D-CNN is used to extract features and an adversarial network is used to self-adaptively align the feature distribution. DeepJDOT uses a 1D-CNN for feature extraction and JDOT for domain adaptation. In WDCNN, DANN and DeepJDOT, the feature extraction component has the same structure, but the domain adaptation is different. In the feature extraction component, DeepJDOT is different from MSCAN-JDOT, but the domain adaptive method is the same. All experimental input data are the same, and the model trained over 100 epochs. Table 4 shows the transfer accuracy under different loads. Figure 7 is a visualization of Table 4, which more intuitively shows the effectiveness of the MSCAN-JDOT model.

#### 4.2.2. Analysis of Fault Diagnosis Experimental Results under Different Loads

As seen from the experimental results in Section 4.2.1, the WDCNN has the worst diagnosis performance, and its average accuracy is 88.25%, and the accuracies for C-A and C-B are 70.96% and 79.44%, respectively. The average accuracy of the CapsuleNet is 91.54%, which is better than WDCNN. The DANN adopts an adversarial network for domain adaptation, and the fault diagnosis performance under different loads are improved to a certain extent. The average accuracy is 92.41%, while the accuracy is only 70.64% for C-A, which is similar to the WDCNN. However, the accuracy is the highest among the four models for B-A. MSCAN-JDOT and DeepJDOT adopt joint distribution optimal transport and demonstrate a significant improvement when compared with other domain adaptive methods. The average fault diagnosis accuracy is approximately 10% higher compared with WDCNN and 6% higher than that of the CapsuleNet and DANN. DeepJDOT uses convolutional networks and joint distribution optimal transport to achieve an average accuracy of 98.25% under different loads and 95.32% for C-A. The proposed MSCAN-JDOT uses a multi-scale capsule attention network and joint distribution optimal transport; this combination improves the fault diagnosis performance most obviously. The average accuracy of MSCAN-JDOT reaches 99.20%, which is the highest among the four models. In addition, the accuracy of MSCAN-JDOT is the highest for A-B, A-C, B-C, C-A, and C-B. It can be proved from the above experimental results that the feature extraction effect of the multi-scale capsule attention network is better than that of the convolutional neural network. The input data use two sets of data for each fault, which equivalently increases the number of fault attributes at different sampling locations. The output of the multi-scale capsule attention network is a vector, which can better extract complex fault features and other fault attributes.

**Figure 8:**
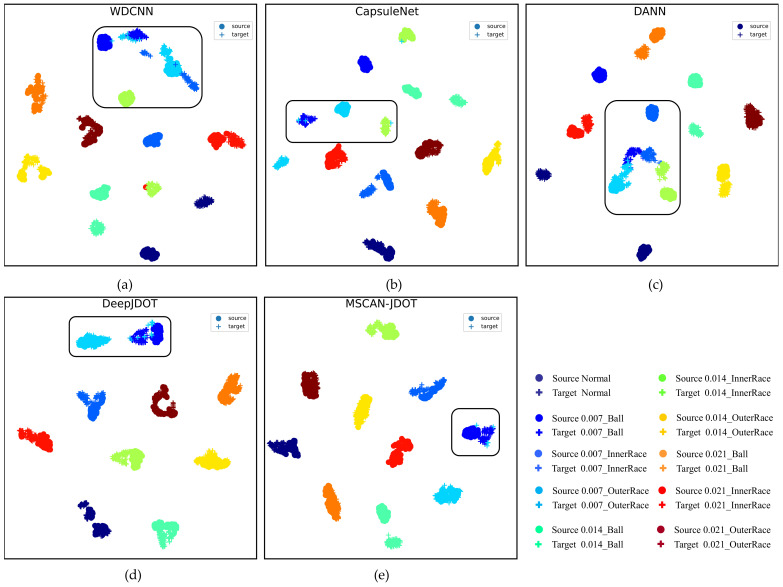
Visualization results of C-A domain-invariant features. Domain-invariant features of (**a**) WDCNN, (**b**) CapsuleNet, (**c**) DANN, (**d**) DeepJDOT, and (**e**) MSCAN-JDOT.

To better verify the performance of MSCAN-JDOT, the features extracted by feature extraction, that is, the domain-invariant features, are reduced to two dimensions using t-sne and visualized. The result for C-A is illustrated in Figure 8, where the source domain data are represented by “·”, the target domain data are represented by “+”, and ten different colors represent ten types of faults. From the data in the boxes in Figure 8a–c, it can be seen that the domain adaptation and fault classification effects of the WDCNN, CapsuleNet and DANN are relatively poor. On the one hand, the distance of features between the target domain and source domain data is sizeable; on the other hand, there is a large amount of overlap between different color blocks. As seen from the data in the box in Figure 8d, DeepJDOT shows some improvement when compared with the WDCNN, CapsuleNet and DANN. The distance of features between the target domain and source domain data is relatively small, and there is less overlap between different color blocks. Figure 8e indicates that the feature alignment effect of the proposed MSCAN-JDOT is similar to that of DeepJDOT, but MSCAN-JDOT has the best fault classification effect, and there is almost no overlap between different color blocks.

**Figure 9:**
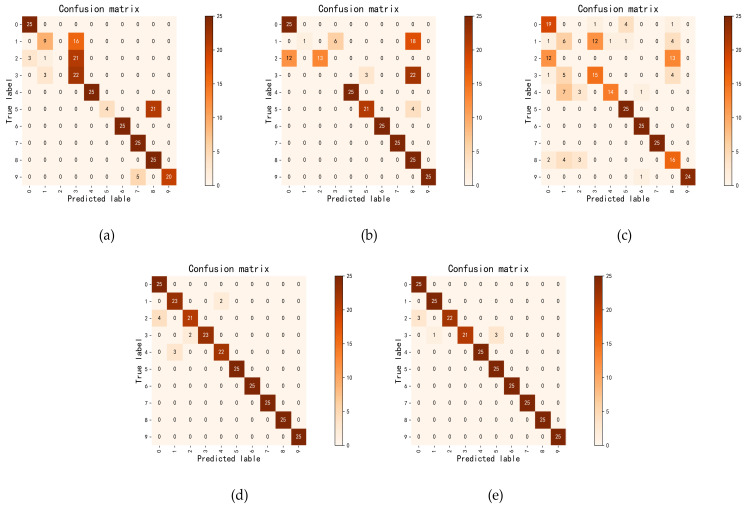
Confusion matrix from C-A test results. Confusion matrix of (**a**) WDCNN, (**b**) CapsuleNet, (**c**) DANN, (**d**) DeepJDOT, and (**e**) MSCAN-JDOT.

Figure 9 illustrates the confusion matrices obtained by different models using the C-A test set, where the horizontal axis is the predicted label, the vertical axis is the true label, and the diagonal elements are the quantity of samples correctly classified, and other positions are the misclassified samples. As seen from Figure 9a–c, the WDCNN, CapsuleNet and DANN have a large number of incorrectly predicted samples. As shown in Figure 9d, the number of incorrectly predicted samples from DeepJDOT is significantly reduced. The confusion matrix of MSCAN-JDOT is illustrated in Figure 9e, from which we can see that the number of incorrectly predicted samples is the smallest. This result indicates that MSCAN-JDOT has the best classification performance, which is consistent with the transfer accuracy in Figure 7 and the t-sne visualization results in Figure 8.

#### 4.2.3. Anti-Noise Experiments under Different Levels of Noise

In this section, MSCAN-JDOT’s anti-noise performance is evaluated. In the actual production environment, noise is inevitable. Hence, Gaussian white noise is added to the raw vibration signal to simulate actual noise. Thus, composite vibration signals are obtained with different signal–noise ratios (SNRs). The SNR is defined as follows:

(16) $${\mathrm{SNR}}_{\mathrm{dB}}=10\log_{10}\left(\frac{S}{N}\right)$$

where S is the power of the raw signal and N is the power of the noise signal. The larger the SNR, the smaller the noise signal. SNR = 0 means that the power of the noise signal is the same as that of the raw signal.

**Figure 10:**
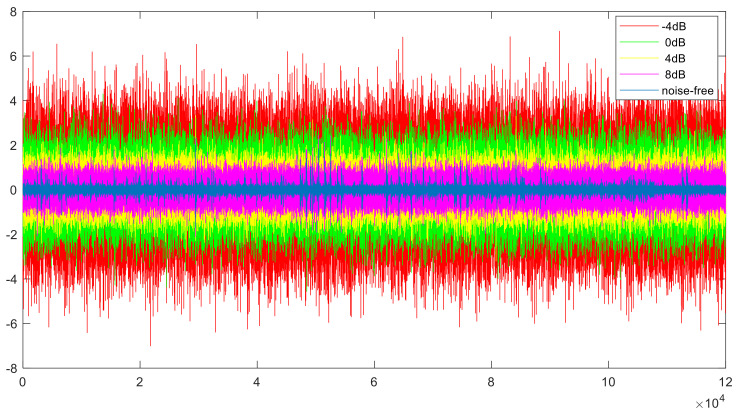
0.014_ball noise-free and noisy data under 3HP.

In this experiment, the anti-noise performance is analyzed using A-C. Noise is added to target domain C while no noise is added to source domain A, and other parameters are unchanged from those used in the fault diagnosis experiments under different loads, which are introduced in Section 4.2.1. The noise-free fault data and noisy data are shown in Figure 10. As shown in Figure 10, with the reduction in noise, the distribution of the noisy fault data becomes increasingly similar to the distribution of the noise-free fault data.

**Figure 11:**
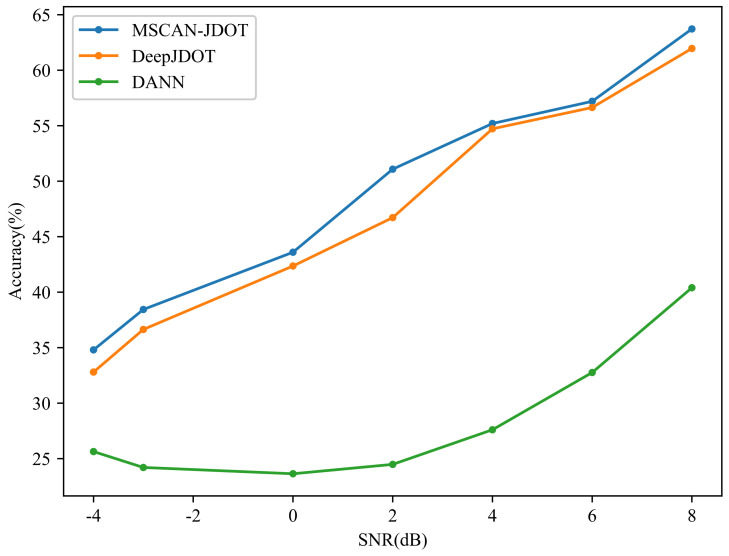
Comparison of A-C transfer results in noisy environment.

Table 5 shows the transfer accuracy from A-C; the signal–noise ratio ranges from -4dB to 8dB. Figure 11 is a visualization of Table 5, and displays the anti-noise performance of MSCAN-JDOT.

#### 4.2.4. Anti-Noise Performance Analysis

According to the results of anti-noise experiment in Section 4.2.3, the proposed MSCAN-JDOT has a higher accuracy compared with other methods under different noise environments. The DANN performed the worst under noisy conditions, with a maximum accuracy of only approximately 40%. Compared with the DANN, DeepJDOT demonstrates a certain improvement in anti-noise performance, with a maximum accuracy of 61.96%. The proposed MSCAN-JDOT achieves 63.72% fault diagnosis accuracy when the SNR equals to 8 dB. Under -4 dB noise, the transfer accuracy of the DANN is less than 30%, the accuracy of DeepJDOT is 32.80%, and the proposed MSCAN-JDOT’s accuracy is 34.80%, the highest accuracy among all tested methods. In the proposed method, the first layer adopts wide convolution kernel and the third layer adopts multi-scale operation. The wide convolution kernel and the multi-scale operation have certain anti-noise ability. In addition, the improved capsule network can better extract multiple attribute features of data by using capsules instead of neurons. Therefore, the features of noise-free data and noisy data can be aligned as much as possible through the domain adaptive module, so as to make the data features in noisy environment more distinguishable. It can also be seen from the results in Figure 11, the anti-noise performance of the proposed method is better than other methods. The above experimental results prove that the multi-scale capsule attention network and JDOT can improve the anti-noise performance. Although the anti-noise ability of the proposed MSCAN-JDOT is enhanced compared with that of other methods, the overall accuracy is not high. This is because MSCAN-JDOT does not add other anti-noise methods.

## 5. Conclusions

To improve fault diagnosis performance for rolling bearings under different loads, this article proposes a transfer learning fault diagnosis method based on multi-scale capsule attention network and joint distribution optimal transport. In this proposed method, the raw, one-dimensional vibration signal is used as input, and the fault features are extracted using the multi-scale capsule attention network. Joint distribution optimal transport is used for fault data domain adaptation under different loads. The proposed MSCAN-JDOT achieves outstanding performance in fault diagnosis under different working loads, with an average accuracy of 99.20%, which is better than that of other transfer learning methods. To address the impact of noise in the actual environment, the anti-noise performance of MSCAN-JDOT is also analyzed in this article. Under seven different noise conditions, the proposed method’s fault diagnosis accuracy is also better than that of other transfer learning methods. The above experiments verify the excellent performance of the proposed MSCAN-JDOT.

In future work, the proposed fault diagnosis method will be further improved, and fault diagnosis under different loads in high-noise conditions will be studied to improve the generalization ability.

## Figures and Tables

**Table 1:** MSCAN-JDOT model parameters.

Layer Name		Kernel Size	Filters	Strides	Padding	Capsule Dimension	Capsules Number	Output Shape	
Input		-	-	-	-	-	-	(2048,2)	
Conv1		64	16	1	same	-	-	(2048,16)	
Conv2		32	32	8	valid	-	-	(253,32)	
Conv3(multi-scale conv)		3/8/16	16/16/16	3	same	-	-	(253,48)	
Attention	avg_pooling	-	-	-	-	-	-	(253,48)	(1,48)
	FC1								(1,16)
	FC2								(1,48)
Primary Capsule		3	256	1	valid	8	32	(32,8)	
Digit Capsule		-	-	-		16	10	(10,16)	
Flatten		-	-	-		-	-	(160)	
FC3		-	-	-		-	-	(128)	
FC4		-	-	-		-	-	(10)	

**Table 2:** Different load datasets of CWRU.

Dataset Name	Speed (rpm)	Load (HP)	Fault Diameter	Fault Location
A	1772	1	0.007,0.014,0.021	Ball, InnerRace, OuterRace
B	1750	2	0.007,0.014,0.021	Ball, InnerRace, OuterRace
C	1730	3	0.007,0.014,0.021	Ball, InnerRace, OuterRace

**Table 3:** Label assignment.

Health Conditions	Label
Normal	0
0.007_Ball	1
0.007_InnerRace	2
0.007_OuterRace	3
0.014_Ball	4
0.014_InnerRace	5
0.014_OuterRace	6
0.021_Ball	7
0.021_InnerRace	8
0.021_OuterRace	9

**Table 4:** Transfer accuracy on CWRU dataset (%).

CWRU	A-B	A-C	B-A	B-C	C-A	C-B	AVG
WDCNN	96.04	92.60	94.68	95.76	70.96	79.44	88.25
CapsuleNet	98.76	99.16	98.16	99.60	73.96	79.60	91.54
DANN	97.20	95.72	98.40	98.12	70.64	94.36	92.41
DeepJDOT	99.72	97.60	98.32	99.08	95.32	99.44	98.25
MSCAN-JDOT	100	100	98.00	100	97.20	100	99.20

**Table 5:** A-C transfer accuracy in noisy environment (%).

SNR(dB)	−4	−2	0	2	4	6	8
DANN	25.64	24.20	23.64	24.48	27.60	32.76	40.40
DeepJDOT	32.80	36.64	42.36	46.72	54.72	56.64	61.96
MSCAN-JDOT	34.80	38.44	43.60	51.08	55.20	57.20	63.72

## Data Availability

Not applicable.
